# Editorial: Trogocytosis and cancer immunity: an intimate and unpredictable relationship

**DOI:** 10.3389/fcell.2026.1975241

**Published:** 2026-09-29

**Authors:** Joyeeta Talukdar, Sorra Sandhya, Bikul Das, Suparno Chakrabarti, Subhradip Karmakar

**Affiliations:** 1 Department of Bio-Chemistry, All India Institute of Medical Sciences (AIIMS), New Delhi, India; 2 Department of Cancer and Stem Cell Biology, Kavi Krishna Laboratory, Indian Institute of Technology Guwahati Research Park, Guwahati, Assam, India; 3 Action Institute of Blood Diseases Transplantation Cellular Therapy (AIBTraCT), Action Cancer Hospital, New Delhi, India; 4 Department of Pharmaceutical Sciences, Dibrugarh University, Dibrugarh, Assam, India; 5 Department of Experimental Therapeutics, Thoreau Laboratory for Global Health, University of Massachusetts, Lowell, MA, United States

**Keywords:** AMPK-SIRT1-PGC-1α, cancer stem cells (CSC), CAR (chimeric antigen receptor), HLA-G, MHC, STAT1-CH25H, trogocytosis, tumor micro environment (TME)

Editorial: Trogocytosis, defined as the rapid, contact-dependent transfer of plasma-membrane fragments and associated molecules between interacting cells, has moved from being regarded as an unusual feature of lymphocyte biology to an increasingly important mechanism of intercellular communication (Kim et al., 2025; Marcarian et al., 2025). In contrast to signalling through soluble factors, trogocytosis allows a receiving cell to directly take in plasma membrane-bound proteins, receptors, lipids, or other species from adjacent cells. The resulting phenotype is then representative of both the cell’s own biosynthetic machinery and its recent physical interactions with other cells (Steele et al., 2016). This is particularly evident during cancer where malignant, immune and stromal cells live in a dense, interacting tumor microenvironment (TME) and are ever-changing under selection imposed by immunity and therapy (Mattei et al., 2022; Kim et al., 2025).

The Research Topic Trogocytosis and Cancer Immunity: An Intimate and Unpredictable Relationship, features four articles discussing topics across the gamut of cancer immunity, cancer stem cell biology, immune tolerance, and mitochondrial quality control. The four articles include a review of trogocytosis and its functions across cancer and immunity (Guha and Banerjee), an original research paper introducing a trogocytosis-active state in colorectal cancer stem cells (Jang et al.), a mini review focusing on HLA-G transfer and immune dysregulation within systemic lupus erythematosus (Karmakar et al.), and a review focusing on exercise induced mitochondria quality control dynamics within aging (Cai et al.). While the later article does not discuss the function of trogocytosis, it presents an overarching view of dynamic regulation of membrane-bound structures as factors in cellular adaptive and homeostatic functions. Viewed together (Figure 1) the articles serve to position trogocytosis within the context of larger studies examining how dynamic membrane behaviour can directly influence cell fate, immunity and cellular identity.

**FIGURE 1:**
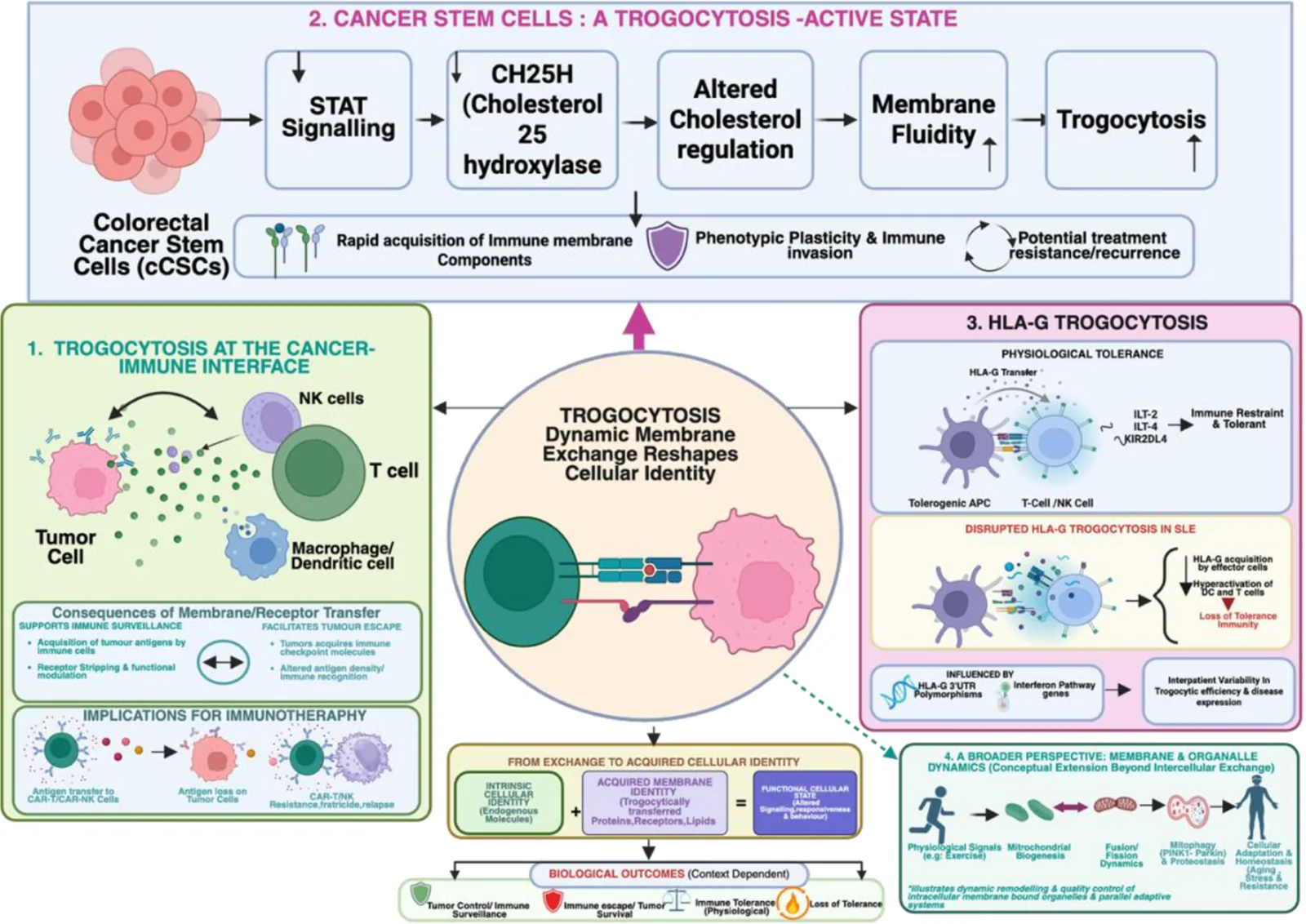
Integrated model of trogocytosis-driven membrane exchange and acquired cellular identity: Trogocytosis at the cancer–immune interface can produce context-dependent outcomes ranging from immune surveillance to immune escape and altered responses to CAR-based immunotherapy. In colorectal cancer stem cells, attenuated STAT1–CH25H signaling is associated with altered cholesterol regulation, increased membrane fluidity, and enhanced trogocytosis. HLA-G transfer illustrates a parallel immunoregulatory role for trogocytosis in immune tolerance and its potential dysregulation in SLE. The lower-right module depicts mitochondrial quality control as a conceptual extension to broader membrane and organelle dynamics; it is not intended to imply a demonstrated mechanistic link with trogocytosis Created in BioRender. 3018, L. (2026) https://BioRender.com/70yz6rj.

Trogocytosis at the cancer–immune interface: Guha and Banerjee provide the conceptual framework for the Research Topic by reviewing the mechanisms, biological consequences, and therapeutic implications of trogocytosis in cancer. They emphasise that the principle of trogocytosis is context-dependent. How the cellular transfer contributes to tumour development depends on the donor and recipient cell type, the mode of transfer, the identity of the transferred cargo, and the biological consequence (phenotype) established by trogocytosis. As a result, trogocytosis cannot be categorised as either tumor enhancing nor tumour-suppressing.

Membrane exchange as such can impact antigen expression level, surface availability of receptors, checkpoint status, and immune cell function inside the TME. Tumour cells can gain anti-immune molecules, whereas immune cells may acquire tumour-associated moieties or modify the targets via receptor ripping and other contact-mediated mechanisms. The bidirectional nature of this crosstalk can be especially important in cellular immunotherapy. In a CAR-T or CAR-NK cell, antigen transfer from tumour cells may serve to reduce target antigen levels on tumour cells while presenting these targets on immune cells, settings which can potentially enhance tumour antigen escape, inducing changes in effector cell phenotype, stimulating exhaustion and/or fratricide. However, immune cell trogocytosis may also serve to suppress tumour activity, and hence the real interest here lies in determining who is doing the transferring of what to whom, under which circumstances, and at what functional impact.

Cancer stem cells as a trogocytosis-active state: Jang et al. advance this framework by asking whether trogocytosis is uniform across malignant-cell states. The authors demonstrated that CSC-enriched populations of colorectal cancer cells exhibited increased T cell membrane-derived membrane content after acquisition compared to CSC-lacking populations. These results linked this therapeutic cell state to a membrane biophysical characteristic. Increased trogocytosis by CSC was associated with membrane ordering defects and enhanced membrane fluidity.

Reduced CH25Hexpression and impaired STAT1 signalling characterised the trogocytosis-active state, whereby the researchers found reduced phosphorylation of STAT1 and reduced CH25Hexpression in the CSC population, whereas inhibition of trogocytosis with IFN—dependent STAT1 signalling selectively expanded the CSC population in the CSC and CSC-lacking spheres. The research found that STAT1 signalling, as observed in the CSCs in single cell RNAseq data from patients with colorectal cancer, was inversely correlated with CH25H levels.

As such, the resulting model—attenuated STAT1–CH25H signaling, altered cholesterol-associated membrane regulation, increased membrane fluidity, and enhanced trogocytosis—providing a mechanistic bridge between cell state, lipid biology, membrane biophysics, and intercellular exchange. This observation matters because CSCs are well known for their tendency towards persistence, plasticity, resistance to therapies, and reemergence. A heightened trogocytic ability would then give these kinds of cells another, genetic-free avenue for promptly changing their surface identity in response to being attacked. As the result does, it moved the field’s efforts beyond the basic question: Do tumor cells indeed pursue trogocytosis, toward that of: Which tumor-cell states specifically afford that capability, and what programs at the biophysical and molecular level permit it?

From membrane exchange to acquired cellular identity: Together, the cancer-focused contributions suggest a useful conceptual distinction between intrinsic cellular identity and acquired membrane identity. Intrinsic identity reflects molecules produced through a cell’s own transcriptional, translational, and metabolic programs. Acquired membrane identity reflects proteins, receptors, lipids, or other membrane components obtained through physical interaction with neighboring cells. In a trogocytosis-active environment, the functional cellular state may therefore emerge from both.

This distinction has experimental implications. Detection of a surface molecule does not necessarily establish that the cell displaying it synthesized it. In tumors, inflammatory tissues, and engineered cellular therapies, investigators may increasingly need to distinguish endogenous expression from trogocytic acquisition. The CSC study adds a further dimension by suggesting that the ability to acquire such an identity is itself regulated by membrane state.

HLA-G trogocytosis and the immune-tolerance axis: Extending the biological relevance of trogocytosis beyond oncology, Karmakar et al. explored regulation of immunity by HLA-G in SLE. The HLA-G non-classical MHC class I molecule possesses immunoregulatory functions. Its exchange through trogocytosis can modify the inhibitory-receptor profile and effector functions of recipient immune cells, highlighting how membrane transfer may propagate tolerogenic properties from cell to cell. Under physiological conditions, HLA-G transfer from tolerogenic antigen-presenting cells to effector lymphocytes engages inhibitory receptors such as ILT2, ILT4, and KIR2DL4 to enforce immune quiescence. In SLE, the authors synthesize evidence that this transfer is disrupted: cytotoxic T cells receive insufficient HLA-G to restrain autoreactivity, dendritic cells depleted of surface HLA-G become hyperactivated, and genetic polymorphisms in the HLA-G 3′ untranslated region, together with variants in interferon-pathway genes, shape interpatient variability in trogocytic efficiency and, by extension, disease heterogeneity. This mini review showed how aberrations in HLA-G expression, trogocytosis, and variation in HLA-G and interferon signaling may generate variability in immune responses to disease. Positioned against cancer literature, this work illustrated the symmetry: while trogocytosis contributes to immune evasion in cancer when favorability to tumor survival is dictated by its efficiency, inadequate immune suppression by aberrant trogocytosis may mediate pathology in autoimmunity.

The trogocytosis paradox: one mechanism, divergent outcomes: Across the Research Topic, trogocytosis emerges as a paradoxical mechanism capable of supporting immune surveillance, immune escape, therapeutic resistance, or immune tolerance. A more informative framework is therefore: donor cell → transferred cargo → recipient cell → acquired phenotype → functional consequence. This directional view moves the field beyond documenting the occurrence of membrane exchange toward defining its causal role within multicellular systems. It also highlights why indiscriminate inhibition of trogocytosis is unlikely to be an optimal therapeutic strategy. Future interventions will probably need to be cargo-specific, cell-specific, and disease-context-specific, with the aim of redirecting rather than simply abolishing membrane exchange.

A broader perspective: from intercellular membrane exchange to organelle quality control: But if trogocytosis demonstrates that material exchange between cells via the plasma membrane dictates how our immunology and tumors fare, then the paper by Cai et al. demonstrates the reciprocal within the individual: quality control of the self by one’s membrane-bound organelles. The authors’ review integrates and delineates components of the mitochondrial quality control (MQC) networks in mitochondrial biogenesis, dynamics of fusion-fission events, clearance via the Pink1-Parkin-mitophagy axis, and the mitochondrial unfolding protein response into an organized process, whose breakdown over time explains reduced bioenergetic and inflammation states in aging. The authors use different training states as illustrative of disparate impacts: endurance training, targeting the network through biogenesis and the fuse through the AMPK-SIRT1-PGC-1α pathway. High-Intensity Interval Training entrenches the system primarily through promotion of the fission machinery and consequent mitophagy and unfolded response; resistance training utilizes the IGF-1/Akt/mTOR pathway in the context of existing network structure to maintain stability rather than promote major biogenesis. The underlying conceit is one’s movement away from a view of membrane-bound entities, whether it is a cell’s outer membrane being physically removed via trogocytosis, or individual mitochondria, a primary membranous organelle, being rebuilt, reformed and recycled through its quality-controlled processes. Both exemplify dynamic adaptive systems, but a direct mechanistic relationship between MQC and trogocytosis is not established by the articles in this Research Topic and remains an area for future investigation.

Translational implications and future directions: The contributions collectively point toward several translational opportunities. First, trogocytosis-associated molecular and biophysical states may have biomarker potential. The CH25H-low, high-membrane-fluidity CSC state provides a candidate framework for identifying tumor populations particularly permissive to membrane acquisition. Secondly, investigating mechanisms responsible for antigen redistribution could allow to better design and follow up the application of CAR-T and CAR-NK based treatments, in cases where trogocytosis contributes to depletion of target antigen density or to modify the behavior of effector cells. Thirdly, the HLA-G example shows the necessity that the manipulation should not lead to the loss of helpful immunoregulatory effects while pathogenic transfer has to be avoided.

Several questions now merit focused investigation. What determines whether a cell–cell contact progresses to productive trogocytosis? Which lipid, cytoskeletal, metabolic, or transcriptional states render specific tumor populations permissive to membrane acquisition? Is the CH25H-low phenotype shared across CSCs from other malignancies? How long do acquired proteins remain on recipient cells, and for how long do they retain functional activity? How does therapeutic pressure alter the direction, frequency, and cargo of trogocytosis?

Technological advances will be central to answering these questions. Live-cell imaging, membrane-specific labeling, lineage tracing, spatial proteomics, lipidomics, and single-cell multi-omics could allow investigators to distinguish endogenously expressed molecules from acquired molecules and reconstruct the direction of membrane transfer in complex tissues. Ultimately, these approaches may enable a “trogocytosis interactome” of the tumor microenvironment: a map linking donor cells, transferred cargo, recipient cells, regulatory pathways, and functional outcomes. Such a framework would move tumor profiling from a static description of cellular composition toward a dynamic description of molecular exchange.

Conclusion: The Research Topic “Trogocytosis and Cancer Immunity: An Intimate and Unpredictable Relationship” highlights how a seemingly simple act of membrane acquisition can produce diverse and sometimes opposing biological outcomes. Guha and Banerjee establish trogocytosis as a bidirectional and therapeutically relevant axis of cancer immunity; Jang et al. identify a CH25H-low, high-membrane-fluidity CSC state associated with enhanced trogocytosis; Karmakar et al. extend the principle to HLA-G-mediated immune tolerance and its dysregulation in autoimmunity; and Cai et al. broaden the conceptual landscape by illustrating the adaptive importance of quality control in membrane-bound organelles.

Collectively, these contributions encourage a view of the plasma membrane not simply as a boundary, but as an adaptive interface through which cellular phenotype can be modified (Miyake and Karasuyama, 2021). In cancer, tumor and immune cells should therefore not always be regarded as fixed participants entering an interaction with immutable identities. Through trogocytosis, the interaction itself can alter the molecular phenotype and subsequent behavior of the participants (Li et al., 2021). The next phase of the field may be defined by four deceptively simple questions: Who gives? Who receives? What is transferred? And what does the recipient become? Answering them may transform trogocytosis from an intriguing cellular phenomenon into a mechanistically defined and therapeutically actionable dimension of cancer immunity.
